# Synthetic Approaches, Properties, and Applications of Acylals in Preparative and Medicinal Chemistry

**DOI:** 10.3390/molecules29184451

**Published:** 2024-09-19

**Authors:** Tobias Keydel, Andreas Link

**Affiliations:** Institute of Pharmacy, University of Greifswald, 17489 Greifswald, Germany; tobias.keydel@uni-greifswald.de

**Keywords:** acylals, diesters, double esters, prodrugs

## Abstract

Diesters of geminal diols (R-CH(O-CO-R′)_2_, RR′C(OCOR″)_2_, etc. with R = H, aryl or alkyl) are termed acylals according to IUPAC recommendations (Rule P-65.6.3.6 Acylals) if the acids involved are carboxylic acids. Similar condensation products can be obtained from various other acidic structures as well, but these related “non-classical acylals”, as one might call them, differ in various aspects from classical acylals and will not be discussed in this article. Carboxylic acid diesters of geminal diols play a prominent role in organic chemistry, not only in their application as protective groups for aldehydes and ketones but also as precursors in the total synthesis of natural compounds and in a variety of organic reactions. What is more, acylals are useful as a key structural motif in clinically validated prodrug approaches. In this review, we summarise the syntheses and chemical properties of such classical acylals and show what potentially under-explored possibilities exist in the field of drug design, especially prodrugs, and classify this functional group in medicinal chemistry.

## 1. Introduction

Many promising drug candidates do not reach market maturity, even though they show good in vitro efficacy at their target and thus represent sought after new treatment options for many diseases. The main reasons for the failure of these compounds during development are safety concerns and lack of efficacy. In the latter case, redundancy in pharmacological networks, short target residence times, fast metabolism and excretion, and many other factors may play a role. However, even simple issues such as poor membrane permeation of charged carboxylate anions can be the reason why substances do not reach their target in vivo, and these pharmacokinetic shortcomings can notoriously cause studies to be discontinued as well [1]. In addition to the personal blows to patients, who are denied hopeful treatment options due to failed drug substances, the financial impact on the research companies is also considerable—after all, today around 10–15 years of development time combined with costs of up to USD 1 billion must be assumed [2]. In order to counter the pharmacokinetic deficiencies of promising drug candidates, prodrug concepts are thus of paramount importance [3,4] in addition to the possibilities offered by pharmaceutical technology in combination with artificial intelligence [5]. In principle, any drug can be subjected to a suitable prodrug concept, whereby each prodrug must fulfil certain requirements, primarily with regard to the risk–benefit ratio, i.e., safety standards within the legal framework apply in the same way as for conventional drug development. In medicinal chemistry, carboxylic acid esters have proven to be a particularly useful tool in prodrug research. The comparatively simple synthesis and the wealth of experience with regard to metabolic behavior in vivo are particularly compelling advantages [6]. However, the esters of other acidic groups, such as phosphoric acid, sulfuric acid, or boronic acids, are also structural components of drug molecules that are associated with similar issues related to charged anionic species that can or must be solved by esterification [7,8,9]. When looking at the legal hurdles for the approval of new drugs and prodrugs, a look at the decisions of the US Food and Drug Administration (FDA) is of interest to many other authorities worldwide. The FDA grants novel drugs NCE (“New Chemical Entity”) status, which is associated with five-year exclusivity periods and thus offers a financial incentive for companies to develop novel drugs. However, this status is not granted to salts or esters of known drugs. This is justified by the unanimous opinion that these derivatives release the parent drug promptly after being introduced into the organism and have no separate pharmacological effect [10]. Nevertheless, ester prodrugs often have a significantly improved effect by influencing pharmacokinetic properties, such as the ability to permeate through membranes or chemical and enzymatic stability [11]. The often reduced solubility of carboxylic acid esters in biological fluids compared to their parent carboxylic acids and, e.g., sodium salts thereof can be cured by the formation of pharmaceutical salts of the obtained esters in many cases, that is, if the drug substance bears other functional groups such as basic amino groups or remaining acidic groups within the molecule that can be exploited for salt formation.

**Figure 1:**
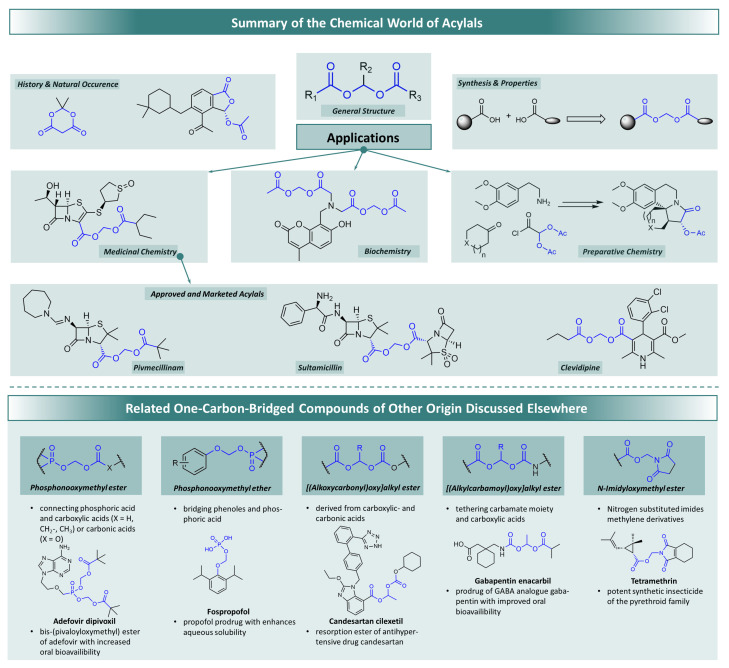
(**Top**) General structure of classic acylals and related chemical aspects discussed in this review. (**Bottom**) Related partial structures from different derivings. Compounds of these types are discussed elsewhere.

Of particular importance in the field of such more demanding prodrug strategies are carboxylic diester compounds of geminal diols, or acylals for short. This structural motif offers the possibility of covalently and stably connecting two acidic substructures via a one-carbon bridge yielding either symmetrical acylals using identical carboxylic acid building blocks or asymmetrical acylals from two different carboxylic acids. The broad availability of non-toxic carboxylic acids thus renders acylals an interesting link to form asymmetrical acylals from drug molecules bearing a carboxy group and various carboxylic acids with great diversity. Figure 1 shows the basic chemical structure in the upper part of the overview. It can be seen that by changing the organic residues, countless compounds with an acylal structure are conceivable and thus a wide range of applications can be covered based on the properties of these compounds. For example, acylals are used in the prodrug research already mentioned—a sub-area of medicinal chemistry. Acylals can also be found in biochemical applications, where they play a particularly important role in imaging processes at the cellular level. On the other hand, certain acylals can be used in preparative chemistry, e.g., in the synthesis of more complex natural products.

The lower part of Figure 1 shows a series of related structures that are also found in many prodrugs, but are not classical acylals. They are derived from other acidic functions, such as phosphoric and carbonic acid, phenols and imides, or from carbamic acid. What they have in common is the rapid hydrolysis in vivo with the cleavage of the parent drug, although a different rationale is followed in each case. It is thus possible to combine lipophilic drugs with a hydrophilic partial structure via a methylene bridge, resulting in increased water solubility and thus intravenous application (a concept, e.g., put into action in the prodrug fospropofol, a phosphate monoester often applied as its highly soluble disodium salt. The increase in oral bioavailability is a formative incentive for the development and use of prodrugs. This can be achieved by converting particularly polar groups of a drug molecule into a non-polar derivative. For example, the phosphonic acid of adefovir leads to an increase in polarity and—conversely—the conversion to alkyl esters leads to a decrease in polarity. In the case of gabapentin, a basic aliphatic amino group giving rise to the formation of polar cations can be masked by incorporation into a carbamate structure. For carboxylic acids such as candesartan, formation of a double ester with carbonic acids, which could be regarded as a triple ester as well, is a common alternative to classical acylals. Since non-polar substances are better able to pass through cell membranes, the masking of polar groups is used to increase the oral bioavailability of drugs by giving them improved membrane permeating ability [12]. The approved prodrugs adefovir dipivoxil, candesartan cilexetil and gabapentin encarbil (shown in Figure 1, bottom) are just a few examples in a large group of similar compounds. They are derivatives of the parent drugs recognizable by their basic name, in which polar groups are coupled with lipophilic groups in acylal-like structures. These examples often have recurring motifs as the lipophilic prodrug part, such as pivalic and isobutyric acid or cyclohexane derivatives [13,14,15,16,17,18]. The non-classical acylal structure in tetramethrin, used as a herbicide applied worldwide, in contrast, consists of a carboxylic acid and an NH acidic imide. This sodium channel modifier causes, like many other herbicides, severe health issues even after inhalation because it is absorbed into the body—an example of the disadvantages of increased oral and inhalative bioavailability [19,20,21].

With a special focus on the applications of acylals in medicinal chemistry and biochemistry, this review gives an overview of historical and nomenclatural considerations of acylals as well. We highlight the synthetic possibilities as well as the application of acylals as synthetic building blocks and would like to summarize a future outlook on this, in our opinion, too rarely considered partial structure.

### Historical Background and Natural Occurrence

**Figure 2:**
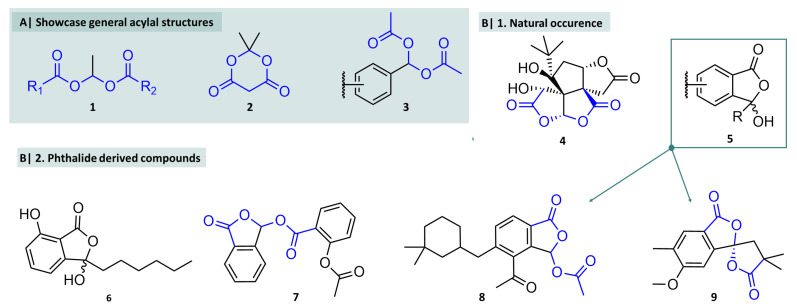
A schematic representation of acylals (**A**) and their natural occurrence (**B1**) as well as the substances derived from them (**B2**).

The first descriptions of these structures date back to the middle of the 19th century when Geuther obtained ethylidene diacetate (Figure 2, compound **1**, R_1_ and R_2_ = CH_3_) while investigating the reaction behaviour of aldehydes with acids. At the beginning of the 20th century, Meldrum reported Meldrum’s acid (Figure 2), which he initially described incorrectly as β-ketocarboxylic acid, but the correct structure was later clarified [22]. Today, this compound plays an important role as a synthetic building block in the total synthesis of natural products and other substances [23,24,25]. From the 1970s onwards, acylals were intensively investigated as a partial structure in prodrugs. The results showed that they were in some cases significantly more sensitive to hydrolysis than simple carboxylic acid esters and were, therefore, used as structural motifs in prodrugs of various drugs, as it was observed that the slow hydrolysis of carboxylic acid esters was associated with insufficient efficacy, e.g., of antibiotics. Acylal structures, on the other hand, hydrolysed significantly faster and thus released the active drug more quickly in effective concentrations. Today, 1,1-diacetates and, in particular, benzaldehyde-derived compounds **3** play a pivotal in protecting group chemistry.

The accidental discovery of ethylidene diacetate (**1**) as a symmetrical acylal, whose synthesis did not require special reagents or extreme conditions, suggests that there may be natural compounds of the methylene or ethylene diacetate type. This assumption is based on the fact that carboxylic acids of various substitution patterns and aldehydes occur ubiquitously in nature. However, to the best of our knowledge, no natural substances of this “linear” type are known.

Nevertheless, some naturally occurring acylals are known, all of which are comparatively complex molecules and the acylal part is incorporated into cyclic structures that also belong to the class of lactones. One known naturally occurring acylal (isolated from the leaves of the ginkgo tree) is bilobalide (**4**), which is contained in various ginkgo preparations and is generally used in treatment of tinnitus [26] or to prevent dementia [27]. A certain efficacy in combination with other drugs has been shown in a mouse model in the treatment of ALS [28]. In addition, certain acylals, namely, esters of hydrophthalide (**5**), appear to be accessible through natural biochemical pathways and have been isolated from various organisms. Figure 2(B1,B2). shows some substances of this class. Fimbricalyxlactone B (**9**), for example, has been isolated from the plant *Strophioblachia fimbricalyx* and is used in folk medicine [29]. Interestingly, the bridging acylal C atom here is also the quaternary C atom of a spiro compound. Luteorosin (**8**), an acetylated hydroxyphthalide, originates from the marine snail *Chromodoris luteorosa* and shows ichthyotoxic activity [30]. Compound **7**, talosalate, is the hydroxyphthalide ester of acetyl salicylic acid (ASS) and is a marketed prodrug for the treatment of inflammation and pain [31]. Building on this particular prodrug approach, several other hydroxyphthalide esters (acylals) were investigated in medicinal chemistry applications, as discussed in the following chapters. The general structure **5** and compound **6** (corollosporine, a metabolite from *Corollospora maritima* with antibacetrial activity) contain a hydroxy group at an anomeric carbon atom at which another, the lactone, oxygen atom is bound. This hemiacetal contradicts a rule of thumb attributed to Emil Erlenmeyer. This rule of thumb for the prediction of stability of molecules is textbook knowledge as *Erlenmeyer-Regel* (German for Erlenmeyer’s rule) in Germany [32]. This rule is not to be confused with the globally accepted Erlenmeyer’s rule on keto-enol tautomerism describing the observation that alkyne hydration does not result in vinyl alcohols, but rather yields the tautomeric aldehydes.

In order to avoid confusion with similar structures, it is essential to create a suitable, unambiguous nomenclature. Especially for the term acylal, for which the IUPAC also provides its own rule (P-65.6.3.6), there are various other formulations in the literature, which makes a detailed literature search difficult. For example, the term “geminal diacetates” has become established for acylals in which both acid components are derived from acetic acid, which is correct according to IUPAC, but is often mentioned in publications without reference to the group of acylals. Another frequently cited term is “acyloxy alkyl ester”, which is not actually incorrect, but is a complication. In the field of prodrug chemistry, certain acid components are found more frequently, whereby a separate name administration has developed for this. For example, pivalic acid is a common structural feature of acylal-containing prodrugs that are often labelled “POM”. This stands for “pivaloyl oxy methyl” and is often placed directly in front of the name of the drug to designate the corresponding prodrug. The chemical precursor, the chloromethyl pivalate, is also often abbreviated as POM-Cl. However, this abbreviation carries the risk of confusion, as “POM” also stands for “phosphonooxymethyl”, i.e., the methyl ester of phosphorous acid and polyoxymethylene, a widely used polymer consisting of formaldehyde or its hydrate as monomer. A standardised designation can, therefore, only be in the interest of chemical research in order to avoid confusion and to be able to draw better boundaries in the event of overlaps. The use of the term diester, which is often used instead of acylals, is inadequate insofar as non-classical acylals also fulfill this designation. The IUPAC recommended term acylal should, therefore, be used consistently.

## 2. Chemistry of Acylals

**Figure 3:**
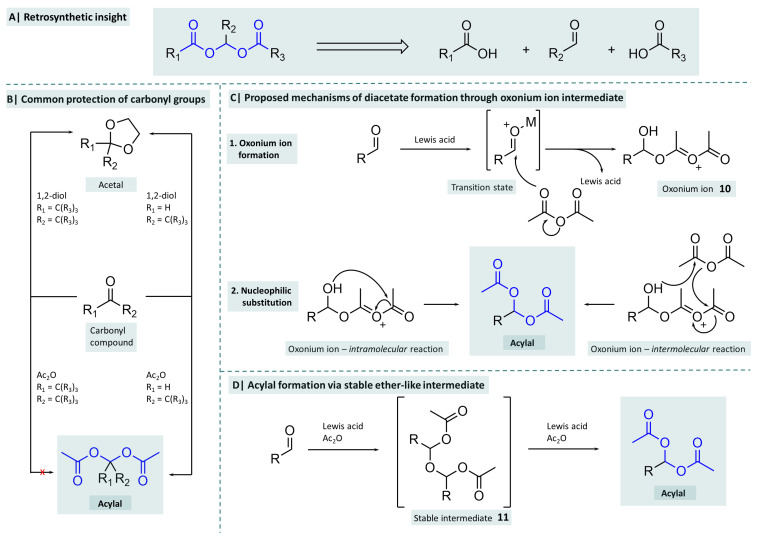
(**A**) Retrosynthetic analysis of an acylal displays its chemical composition of two identical or different carboxylic acids and an aldehyde. (**B**) Common synthetic routes towards carbonyl protecting groups: Aldehydes (R_2_ = H) easily react with anhydrides and vicinal diols to acylals or acetals, respectively. Ketones (R_2_ = org.) react in a similar way with diols to form acetals, but the formation of acylals is only insufficiently possible under the given conditions. (**C**) Formation of an oxonium ion (**10**) by oxidative generation of a positively charged transition state followed by a nucleophilic attack of the anhydride (**1**). Two possible mechanisms, including either intramolecular or intermolecular acetylation to produce the acylal, occur (**2**). (**D**) Formation of isolatable oxybis(arylmethylene)diacetate (**11**) from aromatic aldehydes (R = aryl), proposed to be the key intermediate in 1,1′-diacetate formation.

A retrosynthetic view of the chemical structure of acylals (Figure 3A) reveals that they are condensation products of two carboxylic acids with a hydrate of an aldehyde or ketone (=geminal diols). Due to the reactivity of these carbonyl functions, especially towards nucleophiles or, in the case of aldehydes, the tendency to become oxidised to carboxylic acids, a suitable protective group concept is necessary in the context of preparative chemistry. One of the most frequently used protecting groups for carbonyl functions are acetals (see Figure 3B).

Common acetals are dimethyl and diethyl acetals for which various methods of synthesis have been described [33,34,35]. In addition, cyclic acetals are also frequently used, mostly in the form of 1,3-dioxanes or 1,3-dioxolanes, which can be obtained by reacting the carbonyl group with diols under suitable catalysis [34,36]. Acetals are characterised by their resistance to bases, but in contrast, are unstable in acidic environments. Acylals, on the other hand, are comparatively resistant to acids and are easily hydrolysed by bases. From this point of view, acetals and acylals can be meaningfully incorporated into an orthogonal protecting group concept, which is further reinforced by the fact that the introduction of an acylal function (usually as a diacetate), using the methods described, is selective for aldehydes, while ketones remain unaffected [37].

A mechanistic insight into the formation of diacetates from aldehydes has been part of active research for some time. Kochhar et al. reported that an iron(III) chloride-catalysed reaction of aldehydes with acetic anhydride lead to an inital nucleophilic attack of a carbonyl oxygen of the anhydride on the carbonyl carbon atom of the aldehyde. The resulting oxonium ion **10** then undergoes either inter- or intramolecular acetyl group transfer (see Figure 3). By extending the reaction with asymmetric anhydrides, they assumed from the product distribution that the intramolecular mechanism was more likely [38,39]. Another possibility was given by Yin et al. when they were able to isolate a stable, ether-like intermediate **11** in the reaction of 3-nitrobenzyaldehyde with acetic anhydride in the presence of various Lewis acids. They characterised this product using X-ray crystallography and were able to obtain it in high yields by changing the ratio of equivalents in the reaction. The reaction of this product with Lewis acid-active acetic anhydride yields the acylal expected at the beginning. Since this phenomenon occurs independently of the Lewis acid used, the authors assumed that the formation of the acylal occurs via this intermediate [40].

A decisive characteristic for the introduction of protecting groups is their comparatively simple synthetic accessibility, which is associated with high yields and few by-products, thus reducing the purification effort. Therefore, simple and efficient methods for the synthesis of geminal diesters as protecting groups are of particular interest as well as creating as the possibility for deprotection in a mild, efficient, and chemoselective manner.

### 2.1. Synthesis of Symmetrical Acylals

**Figure 4:**
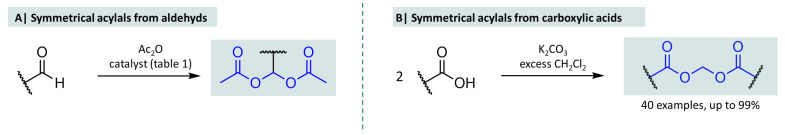
Possible synthetic routes towards symmetrical acylals. (**A**) Aldehydes as starting materials for the acylal synthesis. In this approach, the focus of the desired products lies on the substitution pattern of the aldehydes used. (**B**) Utilisation of dichloromethane as the donator of the bridging carbon atom. Here, the product is a methylene acylal with a variety of carboxylic acid motifs.

The most frequently described route for the synthesis of symmetrical acylals is the reaction of an aldehyde with an acid anhydride under the influence of a suitable catalyst (see Figure 4A). These catalysts are usually Lewis acids and are controlling for both yield and reaction time as well as substrate diversity. In 2020, Rezayati and Ramazani gave a detailed overview of the Lewis acid-catalysed conversions of aldehydes with different substitution patterns to geminal diacetates. A number of different Lewis acids were discussed in their applicability as well as different synthetic approaches, such as solvent-free syntheses or microwave irradiation and ultrasonic treatment. The influence of temperature and solvent properties was discussed as well as approaches to include nanoparticles or ionic liquids in the synthesis protocols [41]. Despite their functionality as catalysts for acylal synthesis, many Lewis acids, especially the inorganic salts, have significant disadvantages, such as hazardous properties [42,43,44], instability to moisture and air, or high price. For this reason, attempts have been made to establish further synthesis methods and, above all, to find catalysts that offer the widest possible range of applications at the lowest possible price and toxicity.

In the early 2000s, Carrigan et al. found that certain bismuth compounds can catalyse the reaction of aromatic aldehydes with acetic anhydride to form the corresponding acylals [45]. They showed that bismuth triflate (Bi(OTf)3·xH_2_O) catalyses a high degree of conversion. The reaction times varied greatly between 10 min and several hours. Ketones remained unaffected by these conditions. Aliphatic aldehydes reacted only very poorly to the corresponding acylals when the reaction took place in a solvent, while the reaction under solvent-free conditions produced acceptable yields. An advantage of many bismuth compounds is their comparatively low toxicity and high efficiency. In further investigations, bismuth nitrate (Bi(NO_3_)3·5H_2_O) also proved to be a suitable catalyst. While the conversion of aromatic aldehydes proved to be similarly good here, aliphatic aldehydes could not be converted satisfactorily even under solvent-free conditions. Nevertheless, bismuth nitrate was able to demonstrate its suitability due to its low cost, stability to air, and nontoxicity [46].

Bismuth-based catalysts can, therefore, be described as substrate-specific for aromatic aldehydes. Based on this, further catalysts could be developed to convert aromatic aldehydes into the corresponding acylals under solvent-free conditions and also to use the catalyst as a reagent to cleave the acylal protecting group in order to recover the parent aldehyde. A suitable reagent was found by Heravi et al. in the form of 12-molybdophosphoric acid (H_3_PMo_12_O_40_), a heteropoly acid. Benzaldehydes with different substitution patterns were completely converted to geminal diacetate in a maximum of two hours. The purified acylals could then be completely converted back into the benzaldehydes in less than one hour using equal molar percentages of catalyst in dry acetone [47].

Various other methods of converting aromatic aldehydes into acylals have been developed. Cerium ammonium nitrate [48] or sulfated zirconium [49] were identified as suitable catalysts. Deka et al. were able to show that, in addition to substituted benzaldehydes, various naturally occurring aliphatic aldehydes can also be converted to the corresponding diacetates in high yields with acetic anhydride in the presence of trimethylchlorosilanes and sodium iodides [50]. Silica-supported perchloric acid also catalyses the reaction of aliphatic aldehydes with acetic anhydride very quickly with excellent yields [51]. A summarising overview of published methods and catalysts in given in Table 1.

Wang et al., who used dichloromethane as a methylene synthon and, in contrast to those mentioned above, were able to dispense with the use of metal-based catalysts, took a different approach (Figure 4B). They synthesised a large number of symmetrical acylals starting from various aliphatic and aromatic carboxylic acids, which they base-catalyzed (potassium carbonate) with an excess of dichloromethane in an aprotic polar solvent (DMSO, DMF, or NMP) and heated for 5 h (130 °C). They all succeeded in isolating the expected products, mostly in excellent yields. They chose the carboxylic acids to be reacted in such a way that the widest possible spectrum of substitution patterns was covered and their results show that only a few structural features have a significant influence on the yields. While yields were satisfying in general, the acylals from α, β-unsaturated carboxylic acids and from α-keto acids were obtained only in low yields under the standard conditions [66].

### 2.2. Synthesis of Asymmetrical Acylals

**Figure 5:**
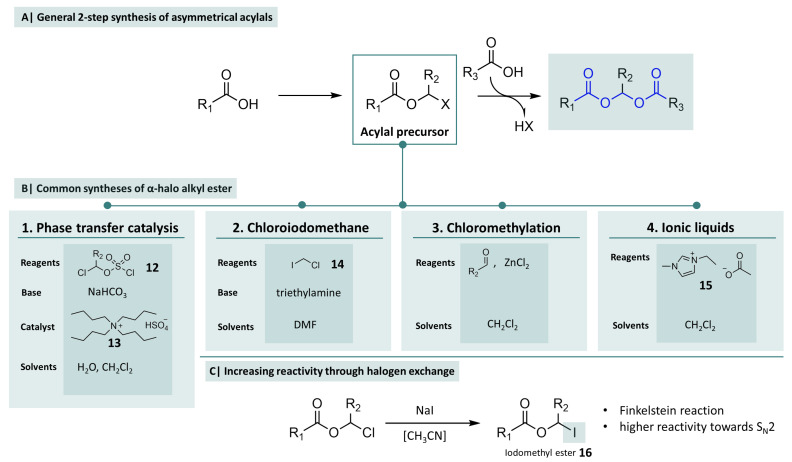
(**A**) General overview on the 2-step acylal synthesis. In step 1, a carboxylic acid is converted into an α-alkyhalide ester. In the second step, the α-alkyhalide ester is reacted in a S_N_2-type reaction with a carboxylic acid yielding an acylal. (**B**) Various methods are described for generating chloromethyl esters. (**C**) In some cases, the chlorine atom can be substituted by iodine in a Finkelstein reaction to enhance reactivity.

As early as the early 1970s, attempts were made to obtain heterogeneous acylals by reacting aldehydes with mixed anhydrides. Despite the use of pure anhydrides, a mixture of different products, including acylals with similar acid residues, was often obtained. In 1973, Scheeren and Tax obtained mixed acylals from the reaction of various acid anhydrides with an excess of formic acid. However, the yields were only moderate and the purification was laborious [67]. A more adequate method is the detour via a synthesis of acylal precursors. The planning of the synthesis focuses on the starting material and requires a conversion adapted to its chemical structure. In Figure 5A, an overview is given of how asymmetrical acylals can be obtained in general. A suitable way to synthesise classic asymmetric acylals is to first convert a carboxylic acid to the corresponding chloromethyl esters with chloromethyl/ethyl chlorosulfate (**12**) in a two-phase transfer catalysis. Quaternary ammonium compounds, such as *tert*-butylammonium hydrogen sulfate (**13**), serve as phase transfer catalysts. In 1994, Harada et al. described this method for the synthesis of chloromethyl esters of N-protected amino acids [68]. This method turned out to be applicable for a large number of carboxylic acids with good yields. This, and the fact that chloromethyl esters are stable and storable, make them good precursors for the synthesis of heterogeneous acylals. Several other methods are described, for example, the utilisation of chloroiodomethane (**14**) as the reagent or the adaption of Blanc chloromethylation conditions. In the next step, the chloromethyl ester and the carboxylic acid react to form the corresponding acylal. This reaction proceeds according to an S_N_2 mechanism and requires an aprotic polar solvent (usually DMF) in the presence of a non-nucleophilic base (TEA, DIPEA, K_2_CO_3_). In order to increase the reactivity of the chloromethyl ester, a halogen exchange can be carried out as part of a Finkelstein reaction. For this purpose, sodium iodide is added to the reaction to form iodomethylesters, such as **16** [69,70].

While the synthesis of symmetrical acylals often takes only a few minutes and the yields are generally very good, which is necessary for a protecting group concept, the preparation of asymmetrical acylals from the precursors mentioned above is more often associated with long reaction times and the use of questionable solvents such as DMF. Therefore, further investigations to optimise these reactions also with regard to principles of green chemistry should be in the interest of academic research as well as industry in order to minimise risks for humans and the environment and to anticipate potential legal restrictions.

### 2.3. Chemical Properties of Acylals: Hydrolysis

If acylals, especially 1,1-diacetates, are considered as protecting groups for aldehydes, simple methods for deprotection are required. The reaction of acylals with bases, such as aqueous NaOH, leads to rapid hydrolysis of the diester structure (saponification), but is not necessarily a suitable deprotection method due to the high reactivity of strong bases, which can lead to various other side reactions. Other chemoselective deprotection methods are listed in Table 1; some catalysts have been shown to be suitable not only for promoting acylal formation but also for deprotecting acylals to give the parent aldehyde. Koszelewski and Ostaszewski were able to show that enzymes (hydrolases) can be used for the deprotection of acylals, while simple esters remain unaffected [71].

**Figure 6:**
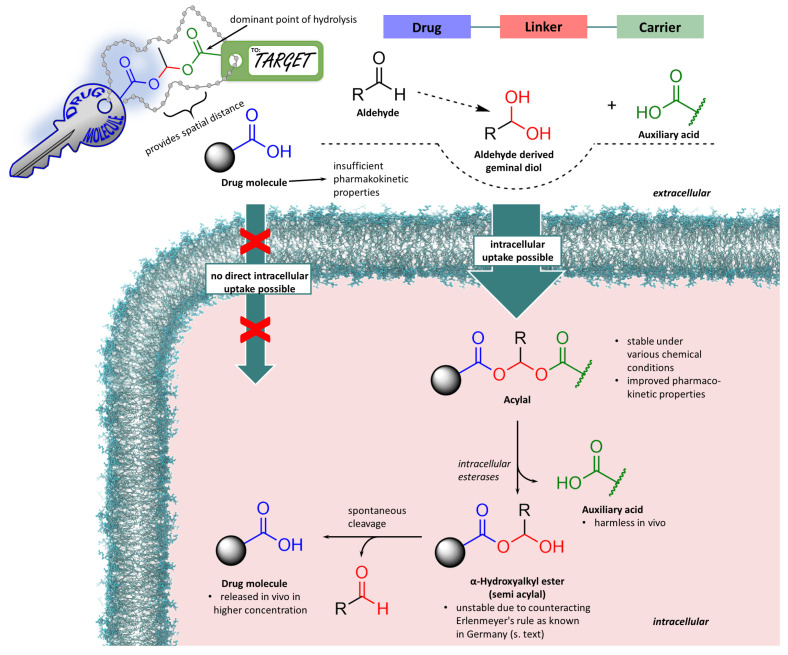
Structural composition of a hypothetical acylal prodrug and hydrolytic behaviour.

Good water solubility of drugs is often cited as a decisive factor for absorption and thus a prerequisite for efficacy [72]. However, good water solubility often goes hand in hand with reduced lipid/water partition coefficients, which has a negative effect on membrane permeation. In the case of carboxy group-containing molecules, their conversion to corresponding esters is a well-established prodrug strategy. This masking of polar groups is not limited to carboxylic acids, but has proven useful for phosphates, phosphonates, and sulfonates as well [73]. About 10% of all commercially available small molecule drugs are used in the form of prodrugs. And of these, almost 50% are converted into their active metabolites by enzymatic hydrolysis. Since hydrolytically active enzymes (hydrolases, EC class 3), including esterases, peptidases, and nucleases, are ubiquitous in the body, it is obvious to pursue prodrug strategies that utilise substrates of these enzymes. Countless alkyl and aryl esters have been clinically trialled and many have been successfully tested and developed to market maturity. Nevertheless, some ester prodrugs did not show the expected oral bioavailability, which is often due to slow or incomplete hydrolysis. It has been shown that this problem can be solved if a double ester prodrug strategy is pursued instead of simple esters. This method is also known as a pro-prodrug approach [3]. Here, the drug molecule is covalently bound to the linker-carrier construct. From a chemical point of view, it is a diester of an aldehyde hydrate (geminal diol), which means that theoretically both ester functions can be subjected to enzymatic hydrolysis in equal measure. Since the auxiliary acid usually has a small, sterically less demanding structure than the drug molecule, it is favoured for enzymatic hydrolysis. After this first saponification, the auxiliary acid and the less stable α-hydroxyalkyl ester of the drug remain, which spontaneously cleaves off the aldehyde and releases the drug molecule (Figure 6).

In order to assess the superiority or inferiority of acylal structures with regard to their hydrolytic resistance to simple ester structures, studies were undertaken in which drugs were converted to ester or double ester prodrugs (an acylal) and their enzyme-catalysed conversion to the parent drug was investigated. It was shown that an acylal prodrug of the loop diuretic furosemide became hydrolysed significantly faster than simple esters of different chain lengths and substitution patterns with half-lives in the range of seconds in rat plasma and liver homogenate, while the equally investigated ester prodrugs showed half-lives of hours or even days [74]. In studies of acyal prodrugs of the gyrase inhibitor nalidixic acid, Roques and co-workers found that there is a correlation between the carbon chain of the prodrug motif attached to the acylal and the stability towards esterases. They were able to show that prodrugs with chain lengths of up to 7 carbon atoms are completely hydrolysed within 24 h. The stability increases with rising chain length, which led to the fact that hydrolysis was not detected within 24 h at a chain length of 15 carbon atoms. Aromatic residues also lead to increased stability, but can also exhibit less favourable solubility behaviour in lipids [75].

## 3. Applications of Acylal Compounds

### 3.1. Acylals in Preparative Chemistry

**Figure 7:**
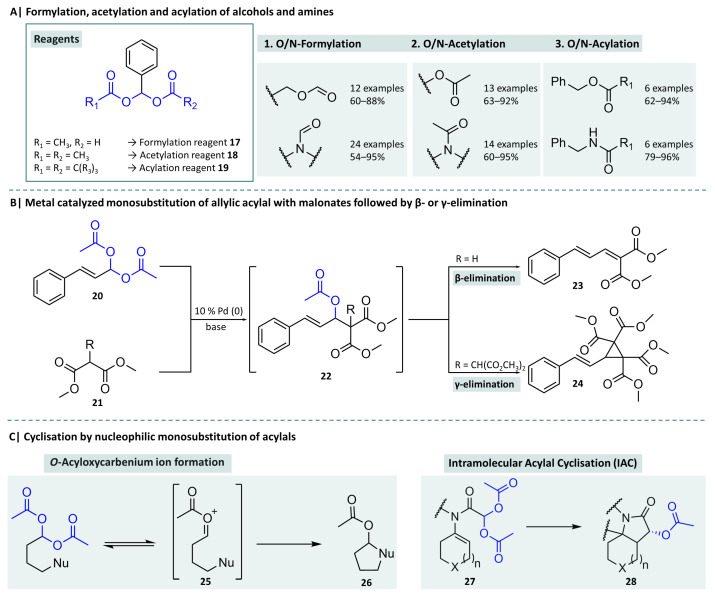
Acylals as synthesis building blocks. (**A**) Application of various benzylidene acylals (**17**–**19**) as esterification and amidation reagents. (**B**) Two-step deacetylation of cinnamaldehyde diacetate (**20**) leads, depending on the substitution pattern, via β- or γ- elimination to conjugated dienes (**23**) and cyclopropane derivatives (**24**). (**C**) *O*-Acyloxycarbenium ion (**25**) as reactive species for intramolecular cyclisation reactions. In an intramolecular acylal cyclisation (IAC), double bonds of unsaturated compounds (**27**) can act as nucleophiles in substitution reactions obtaining new rings (**28**).

The total synthesis of complex natural substances based on simple molecules that are available at low cost has been the focus of many organic synthetic publications [76,77,78,79,80]. Irrespective of the natural product in question, these publications often contain reactions that form carbon-carbon bonds, build up larger rings and ring systems, require oxidations or reductions, derivatise heteroatom-containing partial structures in various ways, or, last but not least, focus on stereochemical considerations. Over the years of developing new synthesic methods, reagents and analytical methods, protecting group concepts and synthesis intermediates have played an important role in the targeted realisation of complex molecules. In this context, the class of acylals has also achieved a special status and several interesting applications for acylals, in particular, 1,1-diacetates, have been described. Acetylations are one of the most frequently occurring chemical reactions not only in the chemistry laboratory but also in living organisms, for example, as post-translational protein modification [81] and, in particular, histone acetylation as a pivotal process in cell cycle regulation [82]. The synthesis of formamides (formylation) is of great interest as they are precursors of many important functional groups, such as nitriles and isocyanides [83,84], or as reagents in important organic reactions, such as the Vilsmeier–Haack reaction [85]. Acetylation and formylation are mainly focused on primary and secondary alcohols and amines to form formates and acetates or formamides and acetamides, respectively. As early as 1941, Hurd, who also introduced the term “acylal” [86], and Green discovered that ethylidene diacetate, which had already been known for some time, reacts with aniline to form acetanilide [87]. They thus showed that the transfer of ester groups is possible with this regard. Certain acylal compounds have proven to be useful tools in this area. Chapman et al. were able to show that symmetrical and asymmetrical acylals are suitable as carriers of acetyl and formyl groups [88,89]. They used formyloxyacetoxyphenylmethane (Figure 7, compound **17**) to insert the formyl group and benzylidene diacetate (**18)** to insert acetyl groups into alcohols and amines. They were able to demonstrate the robustness of their method by achieving good yields even under solvent-free conditions and generating esterification or amide formation even with larger coupling partners using reagents **19**. However, the reagents used in their study were mainly symmetrical acylals, where, in theory, both ester groups can act as the particular acyl group. The authors were able to show that by utilisation of asymmetrical acylals (R_1_ ≠ R_2_) with a sterically demanding substituent (R_1_ = *tert*-butyl), a targeted transfer of the less sterically demanding ester can be achieved.

Another interesting reaction behaviour is shown by diacetates towards dithiols under the catalytic influence of 1-benzyl-4-aza-1-azoniabicyclo[2.2.2]ocatane tribromide (BABOT) in suitable solvents at room temperature. Under these conditions, diacetates react in a transthioacetalisation to corresponding thioacetals, which, in turn, represent important precursors in chemical synthesis [90,91].

The previously described reaction of aldehydes with acetic anhydride under suitable catalysis to the corresponding 1,1-diacetates as an acid-stable protecting group offered the possibility of replacing one or both acetoxy functions using nucleophiles. In 1985, Trost was able to show that different condensation products are formed when the diacetate of cinnamaldehyde (**20**) reacts with differently substituted malonic acid esters (**21**) under palladium catalysis. This initially produces the monosubstituted acetate (**22**), which, depending on the substitution pattern of the malonate, reacts directly to the conjugated diene **23** (when reacting with dimethyl malonate) or to cyclopropane **24** (reaction with dimalonates). The respective yields were in the acceptable to very good range, which may represent progress, since a direct reaction of the aldehyde with the malonate to diene only proceeds with unsatisfactory yields [92,93]. This discovery was further investigated by various research groups, so that Kumar et al. recently published a [3 + 3] heteroannulation of allylic acylals with beta-naphthol derivatives to form chromene derivatives with photochromic properties. Building on this, they used their method to synthesise the structurally related natural product bauhinol D [94].

Condensation products of acylals with bifunctional electrophiles, not only esters but also free acids, amides, ketones, or nitriles, could be generated under indium(III) catalysis, building on the work of Trost et al. Indium(III) chloride served here as a Lewis acid catalyst and is necessary both for the preparation of the acylal from the aldehydes and for their conversion to the condensation products. The acylals are the key intermediates here—a direct conversion of the aldehydes to the desired products could not be detected [95].

Taking a look at the potential reactive centres of a 1,1-diacetate, it can be seen that two points can be considered for a nucleophilic attack. On the one hand, these are both carbonyl carbons of the ester functions and, on the other hand, the carbon atom of the former aldehyde. Investigations have shown that the latter carbon atom is preferentially attacked, which results in the substitution of one of the two acetoxy residues by the nucleophile. Trost was already able to show that the monosubstituted product is detectable. Yadav et al. investigated this behaviour in the hope of generating stable monosubstituted derivatives. They used indium as a catalyst for substitution reactions with allyl bromides. Monosubstituted acetyl derivatives of former 1,1-diacetates were obtained in good yields and fast reaction times (≤5 h). The reactions were specific for monosubstitution without significant amounts of expected by-products, such as aldehydes, pinacols, or homoallylic alcohols, and were able to be carried out in aqueous media [96]. The previously discussed elimination of an acetoxy function from the diacetate leads to the formation of an unsaturated compound, as is common in 1,2-elimination reactions. In this particular case, a highly electrophilic species is formed, the *O*-acyloxocarbenium ion (AOI) (**25**), which is easily attacked by nucleophiles [97]. Using this mechanism, Monaco et al. synthesised various derivatives of the polycyclic Erythrina alkaloids. They started from the cheaply available 2-(3,4-dimethoxyphenyl)ethanamine, which they reacted with cyclohexanone under Dean–Stark conditions to form the corresponding Schiff base. The key structures, the diacetates **27**, were then generated by reacting the imine with 2,2-bis(acetyloxy)acetyl chloride. The now bicyclic cyclohexene derivative reacts under microwave conditions and an excess of boron trifluoride diethyl etherate to the tetracylic products **28** in good yields and, surprisingly, in the form of only one of the four possible diastereomers [98]. They continued their investigations and were able to successfully apply intramolecular acyl cyclisation, as an existential step, to the synthesis of lycoranes, another group of polycyclic alkaloids [99].

### 3.2. A Closer Look at Meldrum’s Acid

**Figure 8:**
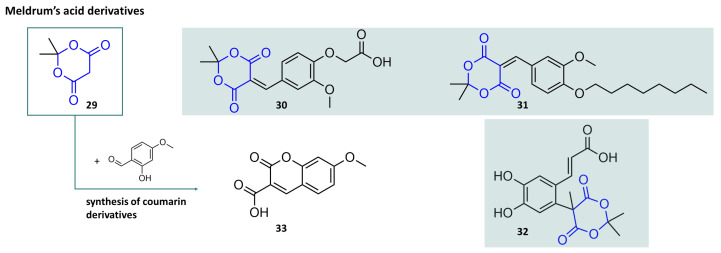
Derivatives of Meldrum’s acid with different pharmacologic effects. In addition to conjugates of Meldrum’s acid with natural compounds, the reaction with 2-hydroxybenzaldehydes obtains coumarin derivatives.

Among the acylal substances used in chemical synthesis, 2,2-dimethyl-1,3-dioxane-4,6-dione, better known as Meldrum’s acid, stands out. This cyclic compound can be understood as a condensation product of malonic acid and acetone and possesses a strong CH-acidity (Figure 8, compound **29**) that exceeds that of the related malonic acid by orders of magnitude. This structural feature makes Meldrum’s acid a reactive compound with regard to a wide variety of reaction types, which have already been extensively summarised [23,100,101,102,103]. Meldrum’s acid and derivatives have proven to be particularly useful in the field of natural product synthesis, for example, as synthetic building blocks for terpene lactones, indole alkaloids, or tetramic acids. As Meldrum’s acid is subject to structural changes in all these approaches, the acylal structure is merely an intermediate. On the other hand, there are studies to characterise derivatives of Meldrum’s acid as pharmacologically active substances. For example, some Meldrum’s acid vanillidene compounds (**30**,**31**) show antimicrobial and antifungal properties [104]. A slight anti-cancer activity was also determined [105]. An antibacterial effect was also found for caffeic acid derivatives (**32**) with Meldrum’s acid moiety [106]. Meldrum’s acid is of synthetic interest as a starting material for the production of coumarin derivatives, secondary plant constituents that are used in a variety of applications, for example, in the perfume industry, but are also toxicologically relevant.

### 3.3. Acylal Compounds in Biochemistry Applications

**Figure 9:**
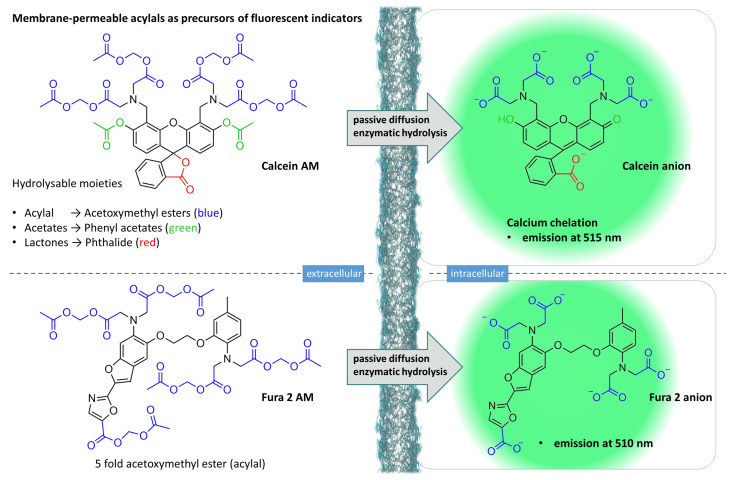
Aminopolycarboxylic acids Calcein and Fura 2, widely used radiometric fluorescent dyes for binding free intracellular calcium ions. Their membrane permeant precursors Calcein AM and Fura-2 AM, containing four and five acetoxymethyl ester substructures, respectively. Cleavage of these groups (in Calcein AM, additional cleavage of phenolic acetates and opening of the lactone ring is required) leads to the particular calcium-chelating reagents.

Its ability to mask carboxylic acid functions in molecules, which, in turn, play an important role in molecules for biochemical applications, and thus influence the physicochemical properties of the entire molecule, makes the diester function an interesting tool in biochemistry. Masking carboxylic acid motifs of a molecule potentially increases its lipophilicity and thus enhance its ability to penetrate cell membranes (vide supra). Since the free carboxylic acid, or the corresponding carboxylate anion, is the actual molecular tool, rapid and complete hydrolysis of the corresponding masking is necessary. In this area, the acylal structure has shown itself to be versatile. Particularly in the field of imaging cell biology, substances have been developed that can pass through the cell membrane comparatively easily in the form of their acylals and are subject to rapid intracellular hydrolysis. Tsien and colleagues have developed various fluorescent dyes, of which Fura 2 has found many applications. Fura 2 AM (AM = acetoxymethyl) is the membrane-passing precursor (Figure 9, lower part). After hydrolysis, the carboxylate ions are trapped inside cells and act as calcium complexators, leading to fluorescence emission which can be detected via fluorescence microscopy [107]. This chemical trick made it possible to observe the movement of calcium ions in living cells for the first time [108].

Building on this breakthrough discovery, a whole series of other substances were developed, many of which contain an acetoxymethyl structure. In most cases, this structure is bound to the carboxylic acids of the actual fluorescent dye (=“acylal”), in other substances to a phenolic hydroxy group (=“hemiacetal ester”) [109,110] or both; acylal and hemiacetal esters can be found in one molecule (e.g., Fluo 3 AM) [111].

An important example of fluorescent dyes is calcein and, in particular, its precursor calcein AM, which is likewise capable of passive diffusion [112]. This non-planar compound, a 4-fold acetoxymethyl ester, additionally carries two acetyl groups on the phenolic hydroxy groups and is present in the lactone form. In this comparatively lipophilic state, the substance is able to penetrate cell membranes. Intracellularly, catalysed by esterases, both the acetoxymethyl ester structures and the phenolic acetyl groups are hydrolysed and the lactone ring is opened leading to calcein, a fluorescent dye with dramatically decreased lipophilicity (Figure 9). In a pH range of 6.5 to 12, it is a versatile tool for detection of several intracellular phenomena [113,114,115].

**Figure 10:**
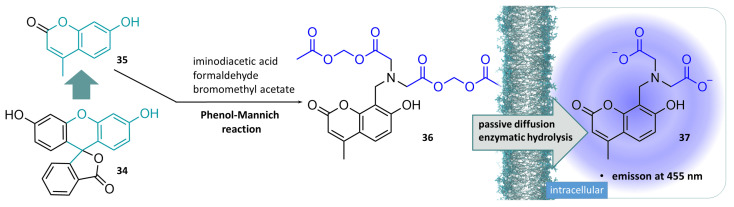
Structural relationship of fluorescein (**34**) and 4-methylumbilleferone (**35**), from which a synthetic approach afforded CalceinBlue AM (**36**) in a one-pot phenol-Mannich reaction. Similar to the above-mentioned compounds Calcein AM and Fura 2 AM, CalceinBlue AM permeates cell membranes followed by esterase-triggered hydrolysis.

A closer look at the chemical structure of calcein shows that it is closely related to fluorescein (**34**). Many fluorescence indicators are based on this structure, and many derivatives have been developed over time [116]. Calcein Blue (Figure 10, **37**), for example, is a structural relative of calcein, which as a cell-penetrating precursor (Calcein Blue AM (**36**)) carries 2 acetoxymethyl ester structures, an optimised synthesis of which was recently published [117]. The authors started from the commercially available 4-methylumbelliferone (**35**), which they synthesised in a one-pot phenol-Mannich condensation with iminodiacetic acid and paraformaldehyde and direct conversion of the in situ-generated caesium salts to the corresponding acetoxymethyl esters. This synthetic approach offers better yields than previously described methods. Similar to the previously described Calcein AM, Calcein Blue AM is carried through the cell membrane by passive diffusion and subsequently cleaved intracellularly by non-specific esterases. The released Calcein Blue chelates calcium ions, resulting in an emission maximum of 455nm, i.e., in the blue spectral range. Other umbelliferone derivatives, including acetoxymethyl ether and esters, have been identified as useful tools in fluorescence-based assays for lipases and esterases. Their applicability could also be demonstrated with regard to high-throughput screening experiments [118].

The translocation property of acetoxymethyl esters of fluorescent compounds, the comparatively rapid hydrolysis by non-specific esterases, harbours the risk of cleavage under certain conditions before the actual site of action, the cell interior, is reached. This problem can occur if there are other physical barriers to overcome in addition to the cell membrane. In plant cells, for example, the cell wall must also be penetrated, which not only consists of polysaccharides such as cellulose, hemicellulose, or pectin, but is also interspersed with other biomolecules, such as proteins [119]. Esterases can also be found in the cell wall, which can cause hydrolysis of the ester structures even before the cell membrane is reached, which is no longer passable for the acidic fluorescence indicators after ester cleavage has taken place [120]. Since ester hydrolysis and diffusion through a membrane are two different processes, namely, of a chemical (hydrolysis) and physical (diffusion) nature, but both processes are dependent on temperature to different degrees in terms of their speed, Zhang et al. have attempted to find a suitable method of suppressing hydrolysis while allowing diffusion to take place without restriction by means of temperature experiments. To this end, they used Fluo-3/AM for calcium detection in wheat roots. They were able to show that incubation of the intact plant material with the acetoxymethyl ester under the normal conditions of the standard protocols resulted in hydrolysis of the esters already taking place extracellularly, which meant that no sufficient concentration of Fluo-3 was produced in the cell. By lowering the temperature (to 4 °C), which results in reduced esterase activity but has little effect on the physical process of diffusion, extracellular ester hydrolysis was dramatically reduced and a suitable intracellular concentration of Fluo-3 was achieved [121].

### 3.4. Acylals in Medicinal Chemistry Applications

The properties of acylals that render them suitable for biochemical applications can also be exploited in the field of medicinal chemistry. So, masking polar carboxylic acids in the form of their corresponding ester is a widely used approach [3]. The possibility of connecting two carboxylic acid-containing molecules via a comparatively small bridge creates a broad spectrum of applications of a pharmaceutical nature. The ease of hydrolysis, which has already been discussed in detail, makes the structure of acylals a very interesting tool in the field of prodrug research [122]. The methylene bridge can connect a variety of different structures, so not only acylals in the strict sense of the IUPAC definition (connection of two carboxylic acids as diesters of geminal diols) can be found in the prodrug design, but also bridges of carboxylic acids with phenols, phosphoric, or phosphonic acid derivatives, sulfonamides, or nitrogen heterocycles, such as tetrazoles, or combinations thereof. This comprehensive addressability of a wide variety of partial structures once again underlines the versatility of the application of methylene-bridged hetero-atoms in the field of prodrug design. A detailed review of some of these related structures has recently been published by Subbaiah et al., albeit the term “acylal” is not contained in the text [4]. Thus, those acylal-related structures that are not defined as acylals in a strict sense are beyond the scope of this article that continues to focus on the classical acylals.

### 3.5. Acylal Prodrugs of β-Lactam Antibiotics

Classical acylal structures are widely used in the field of antibiotic prodrugs, particularly β-lactam antibiotics. Even today, almost 100 years after the discovery of what was later named penicillin by Fleming, penicillins and their further developments are still used as the main drugs for the treatment of a wide range of infectious diseases. The initial euphoria due to their outstanding efficacy and good tolerability was countered over time by the major problem of the development of resistance. This problem is fuelled by the inflationary use of these substances for trivial infections, in mass animal husbandry, and when used incorrectly without a doctor’s prescription, but last but not least, as well by the fact that evolving resistance mechanisms have been an integral part of bacterial life over millennia. An additional increase in the development of resistance has also been observed since the COVID-19 pandemic, which has emphasised the need to search for new active substances using modern technologies [123]. In order to circumvent resistance and address other pathogens, a large number of newer substances have been developed, but well-known substances have also been subjected to various prodrug strategies. Methylene and ethylene acylals are a frequently found structural motif in compounds of the penicillin and cephalosporin class. Both penicillins and cephalosporins share a carboxy function in the beta position to the lactam nitrogen atom in addition to the β-lactam ring that gives them their name. As a derivatisable structural element, this carboxy group offers the possibility of changing the physicochemical properties of the molecule through esterification in such a way that certain pharmacological shortcomings are bypassed.

**Figure 11:**
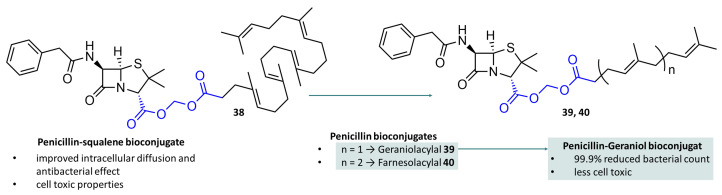
Penicillin G acylal with squalene derivatives forming nanoparticles enhancing pharmacokinetic properties and improving antibacterial effects compared with penicillin G.

The molecular mechanisms that cause the development of resistance are diverse and can be caused by mutations in the penicillin-binding proteins (PBPs), the main targets of β-lactam antibiotics, or by adaptation of the β-lactamases leading to increased metabolisation of the antibiotics, as is the case with *E. coli* [124]. Biofilm formation [125], changes in metabolism [126], formation of efflux pumps [127], or simple overproduction of the addressed proteins also play an important role [128]. Most bacteria occur extracellularly, which simplifies their targeting with antibiotics. However, there are a number of intracellular pathogenic bacteria that pose significant problems worldwide [129]. They are difficult to address, as many antibiotics are characterised by poor intracellular diffusion. Attempts to overcome this problem have been made by Couvreur and co-workers. They developed nanoparticles (NPs) by covalently linking penicillin G with derivatives of squalene (Figure 11), a naturally occurring biocompatible lipid, once via an acetic acid linker with the corresponding alcohol (1,1′,2-trisnorsqualene alcohol) and as an acylal prodrug (**38**) by the reaction of the 1,1′,2-trisnorsqualene chloromethyl ester with penicillin G. Both constructs formed bioconjugates that spontaneously formed stable nanoparticles, and the acylal was shown to be transported into cells by endocytotic processes, including clathrin-dependent endocytosis, where it rapidly and extensively killed the bacteria [130]. However, the use of this bioconjugate was associated with increased cell toxicity, possibly due to the release of squalenic acid, which is highly surface-active and can have a damaging effect on the integrity of the cell. Building on their previous results, they developed further penicillin bioconjugates, for example, with geraniol (**39**) and farnesol (**40**), both polyisoprenes with shorter chain lengths, in the hope of combining consistent transport properties with reduced cell toxicity. While the corresponding farnesol acylal continued to show cell toxicity, the geraniol acylal was able to reduce the number of intracellular bacteria by 99.9% and at the same time showed less cell toxicity. This dramatic improvement in this case also shows the superiority of the acylal diester structure over simple carboxylic acid esters, which were also investigated in this study and did not show sufficient hydrolysis, which was reflected by low levels of free penicillin G and, ultimately, a significantly lower antibiotic effect observed (see Figure 11) [131]. The authors recently published a comprehensive overview of self-assembled lipid-prodrug nanoparticles [132].

**Figure 12:**
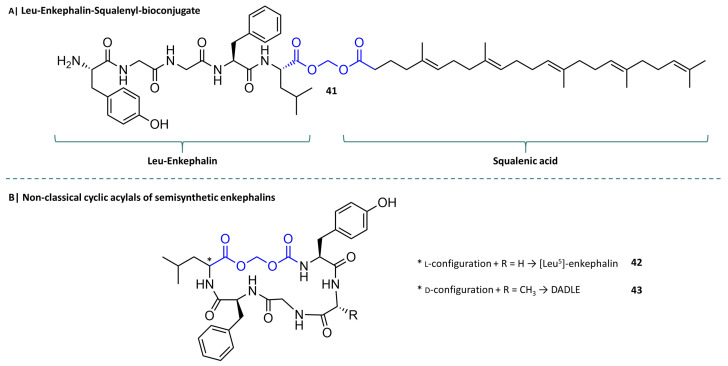
Classic (**A**) and non-classical (**B**) acylals in prodrug approaches of analgetic active pentapetides.

The use of drug–squalene bioconjugates has also proven to be an effective and pharmacokinetically superior prodrug concept in pain therapy research. Building on their findings from the penicillin–squalene bioconjugate experiments, Couvreur’s team has synthesised nanoparticles of squalene bioconjugates with leu-enkephalin (LENK-SA-NPs), an endogenous opioid peptide. In the animal experiment they used, the Hargreaves test, the Lenk squalene acylal (LEN-SQ NP Diox) (Figure 12A, **41**) showed an antihyperalgesic effect that lasted twice as long as that produced by morphine. A prolongation of the paw withdrawal latency compared to the reference times could not be observed. However, the decrease in latency after carrageenan-induced inflammation and the increase in latency after administration of LENK-SQ-NP Diox to the initial level still showed a response in the rats. The authors concluded that Lenk-SQ-NP Diox is not overly effective for acute pain, but can be used for chronic pain [133,134]. A similar approach was taken by Bak et al. using the semisynthetic pentapeptides Leu5-enkephalin (**42**) and DADLE (**43**). They synthesised cyclic prodrugs of both peptides by intramolecularly linking the N- and C-terminus via carbamatoylmethyl groups, an acylal-like structure (Figure 12B) that connects carboxylic acid and carbamic acid via a methylene function. Due to their dramatically altered chemical properties, these prodrugs showed significantly increased permeation in the monolayer uptake assay in Caco-2 cells [135,136,137].

Acylal-based prodrug strategies have proved particularly successful in the field of aminopenicillins. Ampicillin, for example, one of the best-known representatives of the aminopenicillins [138], first described in 1967, has a broader spectrum of activity than other penicillins, but is associated with insufficient oral bioavailability. As early as 1973, a prodrug was described in which the primary amino group and the amide nitrogen atom were condensed with acetone to form the corresponding cyclic aminal [139]. This approach masks the hydrophilic amino group, leading to increased lipophilicity and ultimately to increased oral bioavailability (from 32 to 48%) [122].

**Figure 13:**
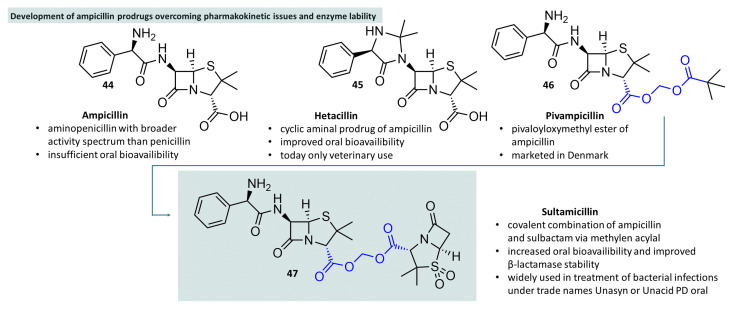
Ampicillin (**44**) and its prodrugs. Most important and widely used prodrug sultamicillin (**47**) is an excellent example of so-called co-drugs.

The approach of increasing lipophilicity by masking polar groups, resulting in increased absorption rates, has proved successful and led to the development of diester prodrugs of ampicillin (Figure 13, **44**). A well-known example of this is pivampicillin (**46**). Because formaldehyde is used for the construction of the methylene bridge, two substituents at this carbon atom are hydrogen atoms and thus no additional chiral center is introduced. This is an advantage as the formation of an additional stereo center would result in the formation of diasteromeric mixtures. This chemical advantage comes with the downside that upon cleavage of this acylal prodrug, the reactive aldehyde formaldehyde that is notoriously toxic when inhaled, is formed inside the patients. The important question, whether this a suitable approach, has been answered by the publication by Dhareshwar and Stella strikingly entitled “Your prodrug releases formaldehyde: Should you be concerned? No!” [140]. The amount of formaldehyde released from double ester prodrugs, such as acylals, can thus be compared to known intake of other sources, such as red wine, and it is thus regarded as of no deeper concern. The risk is especially low if prodrugs are taken only for a week as in the case of antibiotics, even at doses in the range of 1 g per day, or in the case of prodrugs dosed in the mg range even for chronic use. Nevertheless, other less reactive carbonyl compounds, such as acetaldehyde, have been employed in the development of non-classical acylal bacampicillin (1,1-ethylene diester of ampicillin and carbonic acid ethylester). In both cases, the carboxylic acid function has been converted to a geminal diester, on the one hand to a methylene acylal with pivalic acid (pivampicillin) and on the other hand as 1,1-ethylene with ethyl carbonate (bacampicillin). The formation of bacampicillin, however, results in the formation of a fifth stereo center at the 1,1-ethylene acylal bridge giving rise to a nonstoichiometric mixture of diastereomers in the commercially available bacampicillin hydrochloride salt that is an officinal drug substance in Europe (Ph. Eur. 11.6, 0808 (01/2008)) but not in the US. Both diester structures act as resorption esters and lead to dramatically increased oral bioavailability of 86 to 92%.

The covalent binding of a drug to a prodrug motif with the endeavour to ensure that both moieties are restored after in vivo cleavage gave rise to the idea of combining two independently acting drugs. This principle has proven to be successful in the past. Sulfasalazine is a well-known example that was developed over 80 years ago. Chemically, this molecule is a combination of 5-aminosalicylic acid and sulfapyridine in which the aromatic amines are covalently connected via an azo (diazeno) structure. Absorption in the upper small intestine is relatively low at 10–30% and a large percentage of the substance reaches deeper sections of the intestine unchanged. The linker structure is cleaved there by bacterial azoreductases, which leads to the release of pharmacologically active 5-aminosalicylic acid (mesalazine, anti-inflammatory effect) and sulfapyridine (antimicrobial effect). The active ingredient has proven effective in the treatment of chronic inflammatory bowel disease and chronic polyarthritis [141]. This co-drug principle is also found in the acylal prodrug sultamicillin and combines two positive properties of covalent prodrug strategies (see Figure 13, compound **47**). Sultamicillin is a molecule that consists of the β-lactam antibiotic ampicillin and the β-lactamasase inhibitor sulbactam, which are each linked via their carboxylic acid function by means of a methylene bridge. Compared to the two individual drugs, sultamicillin has better peroral bioavailability and thus serves as a kind of transport vehicle for the two polar substances. Once absorbed, sultamicillin is rapidly cleaved into ampicillin and sulbactam, which are acting synergistically as an antibiotic and its guarding lactamase inhibitor, respectively [142,143]. It must be critically stated that this strategy of combining two active principles in one acylal prodrug molecule has a severe drawback: the 1:1 ratio of the two drugs that results from this prodrug approach does not reflect the clinical needs optimally, the dose of ampicillin is too low in comparison to the dose of sulbactam. In other fixed combinations like tazobac, the ratio of a ß-lactam antibiotic and its guarding lactamase inhibitor is 4.6:1, like in piperacillin/tazobactam. This ratio reflects the optimal doses much better. The latter combination of two single active ingredients must be given parenterally, however. It would perhaps have been advisable to combine sultamicillin with another ampicillin prodrug, for example, an acylal prepared from two ampicillin molecules connected via a methylene group in between their carboxy groups, such as the symmetric acylal described by Paternotte et al. [144]. Such a combination could have been optimised to give the clinically most relevant ratio of the two active principles released after hydrolysis.

**Figure 14:**
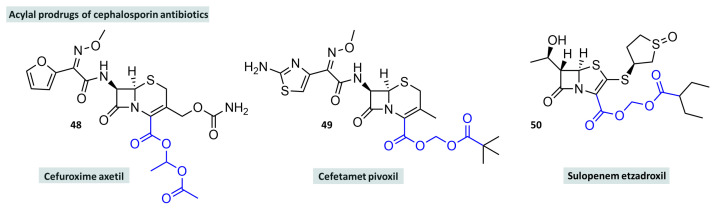
Double ester prodrugs of cephalosporins. Structures of acylal prodrugs of cefuroxim and cefetamet. Cefpodoxim proxetil is a carbonic acid diester prodrug.

The initial, quite understandable, euphoria that accompanied the introduction of penicillins was dampened shortly afterwards, as many patients were no longer responding to the adequate antibiotic treatment due to the emergence of resistance. This led to further developments of β-lactam antibiotics, which culminated in the discovery of cephalosporins, whose basic structure is also of natural origin and whose potency and spectrum of action was improved by partial synthetic changes and which usually exhibit increased metabolic stability [145]. However, the structural similarity to penicillins explains that the pharmacokinetic parameters are similar, so that improved oral bioavailability could not be observed on a sufficient scale. Therefore, appropriate prodrugs are required for sufficient efficacy after oral administration [146,147]. One of the most commonly prescribed cephalosporins and antibiotics in general is cefuroxime. It is used for acute and chronic infections of the respiratory tract as well as the skin and kidneys. It also enters the cerebrospinal fluid—a unique feature among cephalosporins—which makes it possible to treat menigitis with cefuroxime. Cefuroxime is not administered as a free acid. For intravenous administration, the corresponding sodium salt is used, which is highly soluble in water but has little effect when administered orally. Cefuroxime is, therefore, used for oral administration in the form of its resoprtion ester cefuroxime axetil (Figure 14, **48**), an example of an ethylene acylal. The additional methyl group on the bridging carbon atom creates an additional stereocentre, so that cefuroxime axetil is a mixture of diastereomers. This is also used therapeutically as such, as the ester structure has no influence on the pharmacological interaction with the target biomolecule, as it is hydrolysed beforehand. The oral bioavailability of cefuroxime axetil is around 70% [148]. Another example of an acylal prodrug of cephalosporins is cefetamet pivoxil (**49**), a methylene acylal with pivalic acid [149]. Recent developments in the field of carbapenems show that this substance class, which is usually not perorally bioavailable, has sufficiently high oral bioavailability in the form of acylal prodrugs. Examples of this are sulopenem etzadroxil (**50**) and tebipenem pivoxil. Sulopenem is bound to 2-ethylbutanoic acid via a methylene bridge and is currently under review by the FDA for approval for the treatment of uncomplicated urinary tract infections. It is intended to be used in combination with probenecid, which reduces renal clearance and increases plasma levels by inhibiting the organic anion transporter type 1 (OAT1) [150,151]. Cefpodoxime proxetil is a double ester as well but represents a non-classical acylal derived from ethyl carbonate [152].

### 3.6. Further Utilisations in Drug Design

**Figure 15:**
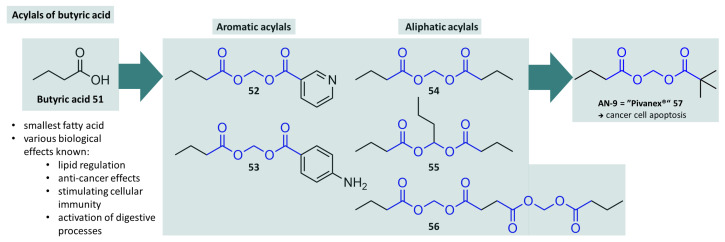
Investigated butyric acid acylal prodrugs with improved properties. AN-9 (**57**) showed good antineoplastic efficacy in vitro in preclinical studies and demonstrated comparable efficacy to known therapeutics in phase II studies in advanced non-small cell lung cancer. The great advantage of this compound is its low toxicity and good tolerability.

In addition to β-lactam antibiotics, which probably represent the largest class of substances that have been extensively subjected to acylal prodrug strategies, such derivatisations can be found in many other drug classes. The obvious reason for this is the presence of many structural motifs of natural origin in drugs which, despite good efficacy at the biological target, exhibit some pharmacokinetic deficits or are metabolised before therapeutic blood levels are reached. For example, it is known from individual studies that the naturally occurring and very simple butyric acid (see Figure 15, **51**) has an inhibitory effect on certain histone deacetylases (HDACs), which are associated with a number of pathological changes, e.g., the development of cancer [153]. However, the treatment of cancer with the administration of pure butyric acid does not lead to the desired effects and would also be limited due to the unpleasant odor. The rapid metabolisation of butyric acid does not allow therapeutic doses to be administered to the site of the biological target molecule, so studies on prodrugs were carried out. Some of these prodrugs, which are to be understood as butyric acid acylals with various other small carboxylic acids, are shown in Figure 15. The best properties in terms of efficacy and pharmacokinetics are shown by AN-9 (“Pivanex”)—the methylene acylal of butyric acid and pivalic acid (**57**). This prodrug has a 100-fold faster effect on cancer cells at a 10-fold lower concentration than pure butyric acid [154,155]. In its capacity as a histone deacetylase inhibitor, AN-9 has also shown efficacy in phase II studies in non-small cell lung cancer (NSCLC), where it was also very well tolerated [156,157]. In further studies (phase IIb), the efficacy in combination with the cytostatic drug docetaxel was investigated and despite initial good results, clinical testing of AN-9 was discontinued by the manufacturer in 2004 due to safety concerns [158]. Nevertheless, many advantages of butyric acids and related butyrates and butyrins (triesters of glycerol) are known and were summarised recently by Gerunova et al. [159].

**Figure 16:**
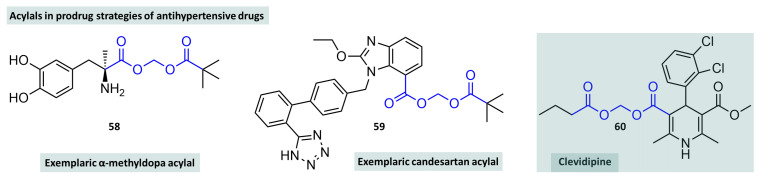
Some acylal prodrugs of antihypertensive drugs.

Hirschmann and colleagues synthesised a series of derivatives of α-methyldopa, an antihypertensive drug structurally derived from dopamine and a methylated derivative of l-dopa. It is mainly used in the treatment of high blood pressure in pregnant women [160]. As a hydrophilic amino acid, methyldopa shows only limited resorption from the gastrointestinal tract. Various ester and acylal prodrugs (Figure 16, compound **58**) were presented, whereby it was shown that the pivaloyloxyethyl acylal was able to lower blood pressure in the animal model (SH rats) three times more potently than methyldopa [161]. In the treatment of high blood pressure, AT_1_ antagonists (“sartans”) are among the drugs of choice, alongside ACE inhibitors. They can also be used to treat cardiac insufficiency or diabetic nephropathy. Candesartan is a well-known representative of this drug class. Since the free acid candesartan has poor membrane permeability, the drug is administered as a prodrug. The blockbuster drug here is candesartan cilexetil—an acylal-type prodrug in which candesartan is combined with a carbonic acid esterified with cyclohexanol via an acetaldehyde linker. In addition to this prodrug, which ultimately reached market maturity, classical acylals were also investigated which showed similar pharmacokinetic behaviour [162]. Another classic acylal prodrug with marketing authorisation is clevidipine (**60**), a substance from the class of calcium channel blockers of the dihydropyridine type (Figure 16). Substances of this type have in common that they carry two carboxylic acid functions in positions 3 and 5 on the eponymous 1,4-dihydropyridine base body, the different esterification of which results in a diversity of substance class representatives. Clevidipine is a methyl ester (position 3) and a butanoyloxymethyl ester in position 5. It is only used intravenously to treat high blood pressure in perioperative situations. The reduction is very rapid and the half-life of the substance is only about one minute [163,164].

**Figure 17:**
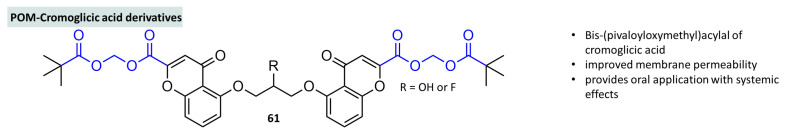
Bis-acylal of cromoglicic acid and its fluoro-deoxy derivative enhance pharmacokinetic spectra.

If absorption via the gastrointestinal tract is not sufficiently possible due to the chemical composition, the use of such a drug is limited either to parenteral application if systemic effects are desired, or to local application. Cromoglicic acid and the related nedocromil, both belonging to the chromone group of substances, which includes the plant constituents flavone and isoflavone as well as the cardioactive substances khellinin and khellin, are used locally to treat acute allergic events and to prevent them. The positive consequence of local application is that systemic side effects are almost completely avoided and the substances are, therefore, well tolerated. Nevertheless, some unexpected effects have been described with systemic application. For example, an inhibitory effect on β-amyloid aggregation and increased microglial uptake and clearance have been observed for cromoglicic acid. Its use in the treatment of Alzheimer’s disease is, therefore, currently the subject of research. Diacylals were presented to increase the membrane permeability of cromoglicic acid and derivatives [165] (Figure 17, **61**).

**Figure 18:**
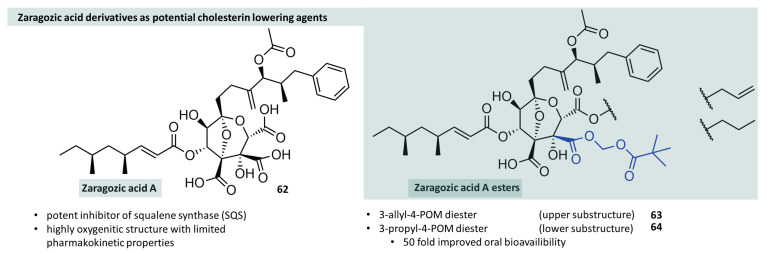
Structural insight on zaragozic acid A and its prodrugs.

Certain ingredients from plants, animals, or fungi can modulate the activity of various enzymes due to their structural similarity to natural substrates. For example, it was found that saragossic acids isolated from the fungi *Leptodontidium trabinellum* and *Sporomiella* species can inhibit squalene synthase (SQS) in the picomolar range [166]. Since squalene synthase catalyzes a key step in the mammalian mevalonate pathway, at the end of which isopentenyl pyrophosphate and dimethylallyl pyrophosphate are the starting materials for human cholesterol biosynthesis, the inhibition of SQS is an interesting target for pharmacological intervention in cases of high cholesterol levels. The in vitro studies on rat liver squalene synthase showed high potency of zaragozic acids (Figure 18, **62**), but in vivo (in mouse and rat) only minor effects could be seen, which was attributed to insufficient oral bioavailability.

**Figure 19:**
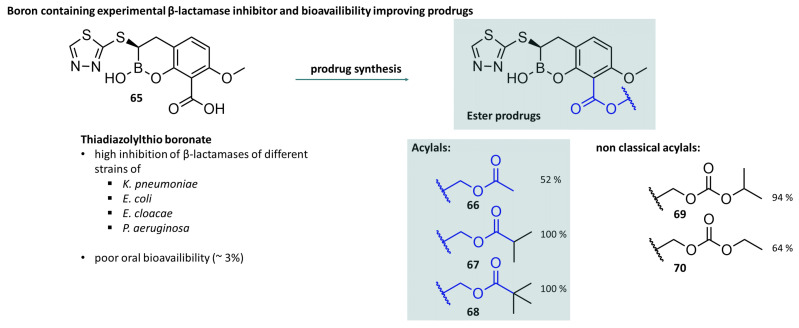
Development of acylal prodrugs of new serine and metallo β-lactamase inhibitors.

Minor modifications to the molecule, mainly involving esterification, resulted in improved oral bioavailability. In particular, pivaloyloxymethyl esters (**63**,**64**) at position 4 were shown to be a useful derivatisation, which, in combination with simple esterification of the carboxylic acid at position 3, resulted in a 50-fold improvement in oral bioavailability compared to the parent acid [167] (Figure 18). Acylal-prodrug strategies are still part of the iterative synthesis of novel drugs commonly used in medicinal chemistry with the aim of positively modelling pharmacokinetics in addition to efficacy at the biological target. Hecker et al. synthesised a series of cyclic boronic acids with inhibitory activity on serine and metallo β-lactamases that can open up new treatment options in combination with known beta-lactam antibiotics (Figure 19, compound **65**). However, as already known for other beta-lactamase inhibitors, these compounds showed poor oral bioavailability. By esterification of the carboxylic acid to acylals (**66**–**68**), the bioavailability could be increased from about 3% to 100% in rat experiments when sterically demanding carboxylic acids were used. The authors also investigated non-classical acylals (**69**,**70**), namely, derivatives of carbonic acid, and were also able to determine a significantly increased, but not 100%, bioavailability [168].

In a recent article, Pivmentel and co-workers have shown how machine learning tools can be used to identify partial structures that can enable molecules to cross the blood–brain barrier by passive diffusion. They investigated a large number of drugs and prodrugs that are known to cross the blood–brain barrier. In the valproic acid prodrug valproate pivoxil, they identified the methylene acylal structure as the driving element for brain passage. Using acylals as the linker structure of a prodrug offers the possibility of delivering even small and partly polar drugs into sensitive physiological compartments, such as the brain, in sufficient quantities despite their poor biopharmaceutical properties [169].

Taken together, acylal prodrug strategies have proven successful in many areas of medicinal chemistry, but were mainly aimed at masking carboxylic acids and thus increasing the lipophilicity of the whole molecule. At this point, we would like to give an outlook on how acylal structures can be used in medicinal chemistry. The principle of linking two pharmacologically active molecules via an acylal bridge, as is the case with sultamicillin, certainly also offers opportunities for other known active substances to bind to structures that serve as recognition motifs for transport proteins. For example, it is known that the human peptide transporter *h*PepT1 transports a whole range of di- and tripeptides from the upper small intestine into the intracellular space [4]. It is also known to transport many peptide-like structures, including drugs. This discovery has already helped several prodrugs with an improved pharmacokinetic profile to reach the market, such as valaciclovir, an l-valine esterified derivative of the antiviral drug acyclovir, in which the amino acid is recognised by the transport protein and active transport of the prodrug is initiated, resulting in significantly increased bioavailability (approx. 55% vs. 10%). Many of these transport proteins, especially those of the SLC family, are interesting targets to enable the transmembrane transport of substances, whereby acylal prodrugs can be trend-setting [170].

## 4. Summary

This review article aims to describe the, in our opinion, underrepresented partial structure “acylal” in a suitable form and to make it visible to a broader field of researchers. To this end, we shed light on the natural occurrence of substances that carry this structure, as well as the first historical chemical laboratory attempts to synthesise it. The use as a protecting group for aldehydes was discussed and the possibilities for synthesis, primarily the progress made in the last 25 years, were shown with the intention of demonstrating the topicality of the use of this protecting group. Acylals also play an important role in preparative chemistry in the synthesis of natural products or in transesterification and amidation. Finally, the importance in medicinal chemistry, in particular, the focus on prodrug applications, which have become very dominant in the field of β-lactam antibiotics, was specifically emphasised, as well as their use in other areas of drug development, where promising substances suffer from pharmacokinetic limitations. Aspects of this pharmacokinetic influence have also been successfully applied in the field of biochemistry and biology and show that acylals can be used ubiquitously and interdisciplinarily.

## 5. Conclusions and Outlook

The wide variety of properties exhibited by substances with an acylal structure makes this group of compounds a very interesting tool not only for the synthetic chemist but also in a very special way for the medicinal chemist. While the use of acylals as protective groups for carbonyl functions has been described in detail and their presentation in terms of substrate specificity and robustness has been presented in a multifaceted manner, some foundations have been laid in the field of medicinal chemistry, but comprehensive investigation as prodrug components has only been undertaken in a few areas. The important safety issue concerning the toxicity of released formaldehyde is still an obstacle for many researchers. However, the notion that this risk is too serious could already have been falsified in the past, but this result, that has recently been summarised by Subbaiah et al. [4], might not have been promulgated enough yet. There is great potential for this partial structure here, as extremely potent active ingredients often suffer from poor physicochemical properties, which could at least be cured to some extent with suitable prodrug strategies. The fact that modern APIs tend to be lipophilic and poorly soluble in biological fluids should not be interpreted as a lack of expertise of medicinal chemists. It is a consequence arising from the necessity of drug substances to leave water shells and diffuse into often hydrophobic binding clefts. In order to do so, the molecular properties necessary are different from the demands during the ADME parts. Thus, it is clear that there will be a continued need to alter these molecular properties temporarily, that is by means of prodrug and salt formation, and one important tool in the medicinal chemists toolbox are acylals of various kinds. The simplicity of the preparation of chloromethyl esters as precursors of acylals should be used as an opportunity to include acylal-prodrug motifs in the iterative synthesis very early in the development of new potential drugs with expected poor pharmacokinetic properties. As a matter of fact, such prodrugs should be synthesised long before a screening compound will be called a prodrug and thus we would encourage medicinal chemists to prepare acylals of test compounds active in enzyme or receptor assays before they are passed on to cellular screening systems—“prohits” rather than prodrugs, technically speaking. In the future, more in-depth investigations are required for all indication areas and, among others, acylals could also represent a suitable object of investigation. 

## Figures and Tables

**Table 1:** Overview of catalysts used in synthesis of symmetrical acylals from aldehydes.

**Catalysts Used for Protection Methods** 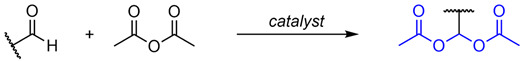					
**Year**	**Catalyst**	**Time**	**Yield (%)**	**Aldehyde**	**Source**
1998	Fe^3^+^^-montmorillonite	10–20 min	81–98	arom.	[52]
2001	Bi(OTf)_3_·xH_2_O	20 min–5 h	64–95	arom./aliph.	[45]
2003	InCl_3_	0.5–6 h	87–96	arom./aliph.	[53]
2004	AlPW_12_O_40_	1–45 min	88–96	arom./aliph.	[54]
2004	Bi(NO_3_)_3_·5H_2_O	1.5–19 h	57–94	arom./aliph.	[46]
2006	InBr_3_	3 min–14 h	76–98	arom./aliph.	[55]
2006	HClO_4_–SiO_2_	1–30 min	90–99	arom./aliph.	[51,56]
2006	C_2_H_6_CuO_6_S_2_/AcOH	1.3–12 h	66–95	arom./aliph.	[57]
2009	SBSSA	2–4 min	72–91	arom.	[58]
2010	see source	2–35 min	45–99	arom./aliph.	[59]
2011	[bmpy]HSO_4_	5–25 min	85–97	arom./aliph.	[60]
2011	CSC-Star	2–7 h	94–96	arom./aliph.	[61]
2014	PSA	4–10 min	93–99	arom./aliph.	[62]
2018	F- or OH-apatites	25 min–48 h ^a^	9–97 ^a^	arom./aliph.	[63]
**Catalysts Used for Protection (Prot.) and Deprotection (Deprot.) Methods**					
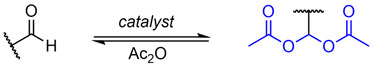					
**Year**	**Catalyst**	**Time** **Prot./Deprot.**	**Yield (%)** **Prot./Deprot.**	**Aldehyde**	**Source**
2002	Zr(CH_3_PO_3_)_1.2_−	12 min–5 h/1–24 h	21–91/32–95	arom./aliph.	[64]
	(O_3_PC_6_H_4_SO_3_H)_0.8_				
2002	CAN	1.5–32 h/4–7 h	70–96/86–91	arom./aliph.	[48]
2006	H_3_PMo_12_O_40_	20–120 min/12–45 min	92–99/92–98	arom.	[47]
2016	Selectfluor^TM^	5–15/15 min	84–98/98	arom./aliph.	[65]

^a^ depending on the composition of the catalyst.

## Data Availability

In this review article no original data is reported. The data presented in this article are taken from the studies and data sets discussed and are cited accordingly.
